# Completing the time trade-off with respondents who are older, in poorer health or with an immigrant background in an EQ-5D-5L valuation study

**DOI:** 10.1007/s10198-022-01517-y

**Published:** 2022-09-02

**Authors:** Tonya Moen Hansen, Knut Stavem, Kim Rand

**Affiliations:** 1 Division for Health Services, Norwegian Institute of Public Health, Oslo, Norway; 2 Health Services Research Unit, Akershus University Hospital, Lørenskog, Norway; 3 Institute of Clinical Medicine, University of Oslo, Oslo, Norway; 4 Department of Pulmonary Medicine, Medical Division, Akershus University Hospital, Lørenskog, Norway; 5 Maths in Health B.V., Rotterdam, The Netherlands

**Keywords:** Health state valuation, Time trade-off, EQ-5D, General public values

## Abstract

**Objectives:**

To determine the effects of age, immigrant background, and poor self-reported health in a general population sample on the probability of non-completion or slow completion of the time trade-off (TTO).

**Methods:**

We used data from an interrupted Norwegian EQ-5D-5L valuation study conducted between 2019 and 2020. All participants responded to background items, irrespective of completion. We used mixed effect logistic regression analysis to assess the effect of old age, poor health, and immigrant background on the probability of non-completion of the TTO, and, for those who completed the TTO, of slow completion times.

**Results:**

First experiences from a Norwegian valuation study were that 29 (5.5%) respondents failed to complete the TTO tasks. For those reporting age over 65 years, poor health, or an immigrant background, 12% failed to complete the TTO. Adjusted odds ratios for predictors of non-completion were statistically significant (age > 65 years, 8.3; EQ-VAS ≤ 50, 3.49; immigrant background, 4.56). Being over 65 years or with an immigrant background also predicted slow completion of both the introduction and TTO tasks.

**Conclusions:**

High age, poor health, and immigrant status increased the risk of not being able to complete the TTO tasks, and of slow completion. Higher non-completion rates and increased completion times suggest that elements of the TTO may be demanding for some respondent groups, with possible implications for representativeness.

## Introduction

The EQ-5D is a generic instrument measuring health-related quality of life (HRQoL), and following recommendations [1], values sets for the EQ-5D are by the far the most used in health economic evaluations to represent societal values [2]. EQ-5D value sets are by convention generated to reflect the preferences of the adult general population of the country in question [3–13], though some value sets have been generated to reflect the preferences of individuals experiencing impaired health [14, 15]. Many countries encourage public participation and recommend the inclusion of societal values in health care decision-making [1]. Inclusive representation and giving a voice to marginalised groups less often represented in research can increase the legitimacy of health care decision-making [16–18], and is supported by initiatives such as INVOLVE in the UK [19]. Many such groups, such as those in poorer health, with immigrant or indigenous background or in older age, may interpret instruments or tasks differently [20], or have different preferences related to health and quality of life than the rest of the population [21–23].

National EQ-5D-5L valuation studies administer time trade-off (TTO) and discrete choice experiment (DCE) tasks using a computer-assisted interview system referred to as EuroQol Valuation Technology (EQ-VT) [24, 25]. Valuation interviews using TTO are demanding, and typically take at least 45–60 min to complete. Some respondents have trouble understanding the TTO task, and increasingly so with age [26], and many respondents value at least one state inconsistently [27]. Consequently, trained interviewers are used to introduce concepts and administer the interview, and participants are recommended to practice using the task as part of an introduction [28]. Risk of cognitive dysfunction and concentration difficulties generally increases with age and in periods of poor health, and respondents with immigrant backgrounds may face challenges when being interviewed in a non-native language. We question whether these characteristics can become barriers for successful completion of TTO interviews.

Using data collected in a Norwegian EQ-5D-5L valuation study, we aimed to investigate these characteristics (age > 65 years, impaired self-reported health, or immigrant background) as potential risk factors for TTO non-completion, and, for those that complete, needing more time.

## Methods

### Study design and participants

The Norwegian valuation study 2019–2020 [29] was interrupted by COVID-19 in March 2020, when 542 of 1300 planned interviews had been conducted. In addition to the 1000 respondents recommended by EuroQol, an additional quota of 300 was dedicated to respondents recruited from healthcare institutions. The sampling strategy aimed to ensure a representative sample of the adult Norwegian general population in terms of geographic region, age, sex, and education. We intentionally contacted respondent groups typically harder to reach or less likely to be included in population surveys, such as those in poorer health, unemployed, ethnic minorities, the elderly, and those with young children.

Sampling was stratified with quotas estimated to reflect the regional composition of age, sex, and educational level. Respondents were recruited at randomly sampled locations within randomly drawn geographic areas in Norway within each region. See the protocol for the Norwegian Valuation study for more information on quotas and location types [29]. Hard-to-reach groups were included by stratified sampling of locations by location type, targeting different respondent groups. Location types such as care homes and activity centres for elderly, Norwegian language learning facilities, social services centres, and primary schools were included to increase representation of previously mentioned hard-to-reach groups.

Contact persons at each location recruited the respondents and were informed of the nature and form of the interviews before recommending the study to potential participants. Contact persons were people employed or otherwise engaged at the sampled location, for example healthcare workers at rehabilitation clinics or care homes, teachers at language learning facilities or managers of sports club. All participants were compensated with a cash gift card (value of ~ 30 Euros) for taking part in the study, irrespective of completion. Contact persons contributed voluntarily and were not compensated. Information materials about the study and participation in the interviews were provided to contact persons and respondents prior to scheduling the interview.

The Regional Committee for Medical and Research Ethics reviewed the protocol for the Norwegian Valuation study and stated that their approval was not required. The Norwegian Institute of Public Health approved the Data Protection Impact Assessment for the study on the 30th of September 2019.

#### Interviews and data collection

Trained interviewers (*n* = 13) conducted face-to-face interviews at 45 sampled locations in three different regions of Norway, administering time trade-off (TTO) and discrete choice experiment (DCE) tasks using the EuroQol Portable Valuation Technology (EQ-PVT), a PowerPoint based version of the EQ-VT software.

Using TTO, respondents indicate their preference between two competing scenarios involving a shorter life in full health and 10 years in a poorer health state; trading away years of life in full health in an iterative process until a point of preferential indifference between the two scenarios is reached. Following current EQ-VT protocol, respondents are administered 10 EQ-5D-5L health states for valuation using TTO, as well as 7 DCE tasks, and a paper questionnaire describing their own health and background, including age, self-reported health and immigrant background which were considered relevant as proxy measures for the selected concepts.

The software automatically collects completion times per task. Interviewers guided respondents through each part of the interview unless the respondent wished to conclude the interview partially or completely. All respondents completed a paper questionnaire describing their background, irrespective of TTO completion. Despite allocation of at least 90 min per interview plus breaks, interviews were completed without time constraints. Interviews were continued even if they took longer than the allocated 90 min.

### Statistical analysis

The analyses were based on data collected for a valuation study, and thus variables included in the analyses were selected as proxy measures for poorer cognition, ability to concentrate over longer periods of time, or language barriers. Respondents described themselves based on a range of survey items collected in the paper questionnaire. Of the collected variables, age, self-reported health, and immigrant background were most relevant as proxy measures for the selected concepts. Dummy variables indicating immigrant background (born in Norway vs. born outside Norway), poor health (self-reported EQ VAS scores ≤ 50 vs ≥ 50), old age ( ≥ 65 years vs ≤ 65 years), higher education (university level vs. lower level) were used in the analyses. Predictors were assessed in both univariate and multivariate analyses.

Predictors of non-completion and slow completion of the TTO part of the interview were assessed using mixed effects logistic regression. For those that completed the TTO, slow completion was defined as completion time in the upper quartile in the total sample, i.e., > 12.7 min for the introduction and > 16.4 min for the following 10 TTO tasks. Results from the multivariate analyses are presented as adjusted odds ratios (OR).

Models for both TTO non-completion and time use controlled for interviewer effects by including a random intercept at the interviewer level. For slow completion times, sensitivity analyses were performed including dummy variables for each interviewer.

R version 3.6.2 was used for the statistical analyses [30].

## Results

### Sample characteristics and completion of time trade-off

Of the 542 respondents who started an interview, 8 were excluded due to missing data or retracted consent. Of the remaining 534 respondents, 505 completed the TTO part of the interview. In comparison, 522 respondents completed the DCE tasks and only one respondent failed to complete both the TTO and the DCE. Where there were violations of defined data quality standards (*n* = 17), these were primarily for an inconsistent valuation of the worst possible health state, i.e., utility more than 0.5 than any other health state. Of non-completers of the TTO, more than half were over the age 65, 28% reported a VAS score of 50 or less, and 38% had immigrated to Norway (Table 1). Among those not completing the TTO, half of those reporting poor health were also over the age of 65 years, whilst only 25% of those with poor health in group that completed were also over 65 years. Of those reporting either age over 65 years of age, an EQ VAS score below 50 or immigrant background 12% did not complete the TTO.

**Table 1:** Demographics and EQ VAS score prior to the time trade-off task, p-values from two-sided t-tests for sample means, and z-tests for sample proportions using a significance level of 0.05

	Incomplete TTO	Completed TTO	
*N*	29	505	*p*
Age, mean (SD)	60.2 (19.9)	44.6 (16.7)	< 0.001
No. over 65 years of age (%)	15 (52)	67 (13)	< 0.001
No. of women (%)	18 (62)	300 (59)	0.764
No. with higher education (%)	13 (45)	294 (58)	0.159
No. with immigrant background (%)	11 (38)	98 (19)	0.016
EQ VAS score, mean (SD)	68.5 (24.0)	78.5 (16.4)	0.002
No. with EQ VAS < = 50 (%)	8 (28)	47 (9)	0.002

Adjusted OR for predictors of incomplete TTO (adj. OR: age > 65: 8.3, EQ-VAS ≤ 50: 3.49, immigrant background: 4.56) were notable and statistically significant (Table 2).

**Table 2:** Predictors of non-completion of the time trade-off and slow completion of the introduction to the TTO and the 10 following TTO tasks, mixed effects logistic regression with a random intercept at the interviewer level, presented as adjusted odds ratios

	Incomplete TTO (*n* = 534)		Slow introduction^a^ (*n* = 505)		Slow TTO task completion^b^ (*n* = 505)	
	adj. OR (95% CI)	*p*	adj. OR (95% CI)	*p*	adj. OR (95% CI)	*p*
Over 65 years of age	8.30 (3.58, 19.24)	< 0.001	4.96 (2.68, 9.17)	< 0.001	3.13 (1.76, 5.57)	< 0.001
EQ VAS under 50	3.49 (1.35, 9.04)	0.011	2.11 (1.03, 4.32)	0.043	1.83 (0.93, 3.61)	0.080
Immigrant background	4.56 (1.87, 11.14)	0.001	1.91 (1.09, 3.34)	0.024	1.83 (1.09, 3.07)	0.022
Higher education	0.63 (0.28, 1.40)	0.257	0.87 (0.54, 1.38)	0.54	0.82 (0.53, 1.27)	0.37

*CI* confidence interval

^a^Slow completion of introduction: > 12.7 min

^b^Slow TTO task completion: > 16.4 min

### Time to complete time trade-off

**Fig. 1:**
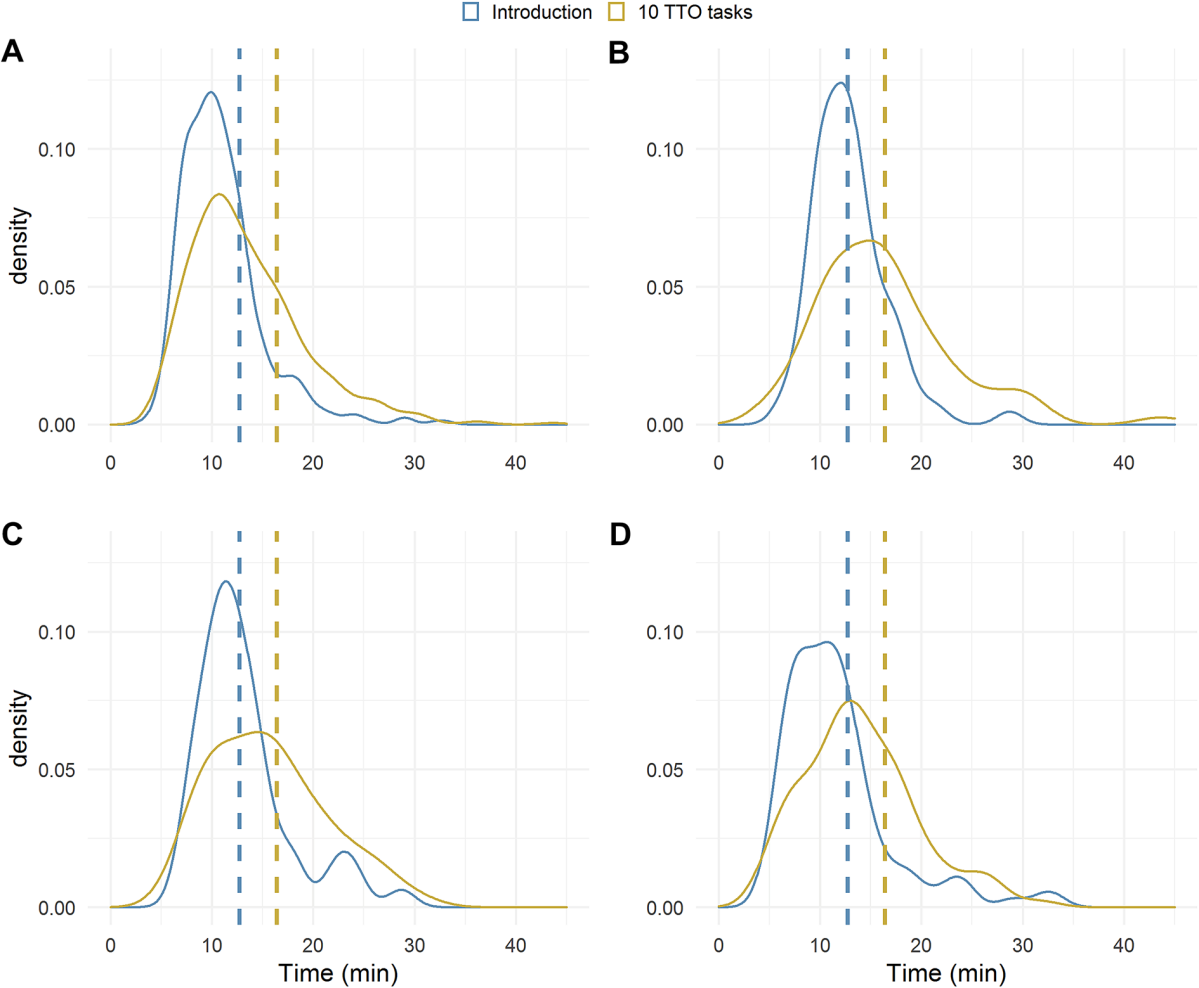
Completion time for introduction and following 10 TTO tasks for **A** All respondents, **B** Respondents over the age of 65, **C** Respondents with EQ VAS score below 50, and **D** immigrants. Dashed line indicates upper quartile of time to complete in the total sample. Time in minutes

For those who completed the TTO, the introduction took median 10.2 min (range 3.5–33.1), whilst the 10 following tasks took median 12.3 min (range 4.2–43.7; Fig. 1). Using the upper quartile of completion times in the sample to define slow completion, 25% of respondents were slow completers of either the introduction or the ten TTO tasks.

Of the tested characteristics, those reporting age over 65 years and those reporting immigrant background were more likely to be slow completers (completion time in upper quartile) of both the introduction and the 10 TTO tasks (age > 65: *p* < 0.001, immigrant background: *p* < 0.05) (Table 2). Those reporting an EQ VAS score under 50 were more likely to be slow completes of the introduction (*p* < 0.05).

Findings were robust in repeated analyses which controlled for the interviewer administering the interview as a fixed effect using dummy variables.

## Discussion

We hypothesized that TTO tasks may be too challenging for some groups, leading to higher rates of non-completion or slow completion. Although most respondents over 65 years of age, with poor self-reported health, or with an immigrant background were able to complete all 10 TTO tasks, all these three characteristics were strong predictors of non-completion. Of those that completed, the same characteristics were predictors of increased time use. Inference from the observed differences in time use for the TTO is, however, open to interpretation; individuals struggling with a task may not necessarily spend more time, as some may be inclined to rush through to complete the task quicker, while highly engaged respondents may take their time to contemplate and value each health state presented.

Drop-out rates in valuation studies among older respondents and those in poorer health have been noted previously, for example described in the development of the methods used today [31]. Few published EQ-5D-5L valuation studies conducted using EQ-VT (2012 and onwards) elaborate on any difficulties respondents may have had completing the task beyond commenting that those with cognitive impairments, or who struggled to comprehend the task were excluded. One study mentioned the need for additional visual aids to help elderly respondents concentrate on the task who “often felt tired after spending a long time working at a screen in the composite TTO tasks”, resulting in lack of focus and increased likelihood of random responses.

Respondents’ own evaluation of task difficulty varies [26–28]. Previous studies have presented findings suggesting that the tasks are easily misunderstood and are difficult to complete for many respondents [26, 27]. Increased drop-out in some groups is another indication that the posed tasks could be too demanding for some. The role of the interviewer has been shown to be important for respondent engagement and completion [28, 32].

As the number of available value sets and their uses increase, attention to validity of the measures and issues with legitimacy has also increased [33–36]. There is a growing emphasis on the representativeness of value sets and inclusion of politically and empirically important subgroups of the population [16, 37, 38], to ensure legitimacy in the setting in which they are used and comparability between value sets. To date, most valuation studies use sampling strategies to mirror the adult general population in terms of age, sex, and educational level [3–13], at times also socioeconomic/employment status, and religion or ethnicity. In several fields of research, inclusion of minority groups has become a requirement, as well as inclusion across all age groups. For instance, the US National Institutes of Health made the inclusion of minority groups and women in all sponsored clinical research a requirement in 1993, and more recently, inclusion across the lifespan [39].

Where identifiable subgroups of respondents face barriers to task completion, seeking to include a representative sample of the population may prove insufficient, even when included. Mentally demanding methods, such as the TTO, can result in increased non-completion or random responses in specific respondent groups. For example, older respondents more often needed more time or failed to complete the TTO tasks. If values are to be representative of age, all age groups should be equally able to complete. In the present study, individuals more likely to have hands-on experience with ill health were also at greater risk of not completing the TTO tasks. The complexity of the TTO can be a real barrier for elicitation of health state values from vulnerable groups, such as those with dementia [22] and younger respondents (adolescents and children) [40]. Valuation of health states using other methods, such as Discrete Choice Experiments (DCE) and Best Worst Scaling, are arguably easier to understand conceptually and have been applied successfully in these same respondent groups [41, 42]. Despite collecting both TTO and DCE responses, as is now recommended by EQ-VT protocols for EQ-5D-5L valuation studies [24], several EQ-5D-5L value sets were still estimated based on TTO responses only [3, 4, 43, 44], often stating poor agreement between DCE and TTO preferences [45, 46]. The extent to which these two methods tap into the same underlying preference structure remains uncertain, and whether they can be used interchangeably is therefore controversial.

### Strengths and limitations

The data included in the analyses were collected as part of a national valuation study complying to EQ-VT protocol v2.1 [24], and thus a realistic setting for use of the TTO in a general population sample. Interviewers were trained according to protocol, and reviews of data quality throughout data collection suggested that interviewers were performing well and consistently. Despite being conducted in a single population, the aspects of the task and administration of the interviews are standardised between valuation studies, and thus, findings can be generalised to other studies following similar protocols.

The sampling strategy was specifically designed to increase participation of hard-to-reach groups in the population, such as those in poor health or with an immigrant background. Local contact persons at each location cost-effectively recruited respondents for the study, and because contact persons were employed at or otherwise associated with each sampled location, they were often familiar with potential respondents and were able to assess whether they were suitable for participation based on the information about the study they had received beforehand. This was particularly useful when recruiting more vulnerable groups such as elderly, those with experience with poor health, and those with an immigrant background. For example, one of the predefined location types per geographic area was Norwegian language learning facilities, targeting respondents recently immigrated to Norway. Contact persons at these locations were able to assure a minimum level of Norwegian language proficiency among respondents. Around one third of respondents reporting an immigrant background were recruited from these facilities (*n* = 34). Contact persons received no compensation for their assistance recruiting respondents, and thus had no personal incentives to recruit further respondents beyond than those willing to participate.

The chosen recruitment strategy also means that included respondents were not chosen completely at random and had already been deemed suitable for participation. If we interpret our findings as the TTO task itself imposing a barrier for completion, we might expect even higher non-completion rates when applying a sampling and recruitment strategy that more randomly and directly sampled and recruited respondents.

When data collection was abruptly stopped some interviewers had completed substantially more interviews than others. Our experience was also that despite training and use of an extensive interview guide, respondents often had other questions related to the task, and some degree of interviewer effects in the handling of different situations was inevitable, possibly also in the case of dropping out of the interview. We attempted to control for such interviewer effects in the analyses by including a random intercept at the interviewer level.

Another limitation was that the EQ-PVT software did not easily allow collection of data from partially completed interviews. When respondents indicated or were considered unable to complete, interviewers had the option of closing the TTO interview (resulting in loss of partially complete TTO data), or to quickly complete the remaining tasks, providing a response which would later need to be removed or flagged in the feedback module, which would require a substantial number of clicks. No response was not an option. Consequently, partial completion responses from the TTO part of the interview were not saved, and information from these interviews, for example indicating at which point in the interview respondents typically drop out, and whether their responses prior to dropping out differed from other respondents in their population strata, were not available for analysis. Also, because the data were collected with the primary objective of valuing the EQ-5D-5L in a general population, other potentially relevant measures for the objectives of this study, such as respondent or interviewer evaluations of the interview, were not collected.

All three respondent characteristics included in the analyses (age > 65 years, EQ VAS < 50, born outside of Norway) represented other not measured phenomena, acting as proxy variables for higher probability of poorer cognition and ability to concentrate over longer lengths of time or possible language barriers. In particular, the proxy measure for possible language barriers, i.e., immigrant background, is crude and presumptive. Whilst reporting ‘not born in Norway’ arguably increases the probability of having a language other than Norwegian as their primary language, language proficiency will be affected by other factors, such as duration of stay in Norway and education. Amongst those newly immigrated to Norway, language proficiency will also likely vary substantially.

## Conclusion

More than one in ten respondents indicating age over 65 years, immigrant background, or poor health failed to complete the TTO in a valuation study for the EQ-5D in a Norwegian general population. Respondents with these characteristics that completed the TTO also required more time. Higher non-completion rates and time use in specific groups can be costly but can be accounted for in the planning of valuation studies. The results can suggest that elements of the TTO may be more demanding for some subgroups of respondents, possibly leading to those able to complete not being fully representative of their relevant population strata.

## Data Availability

Raw data cannot be shared due to privacy laws in Norway.

## Abbreviations

DCE: Discrete Choice Experiment
EQ-VT: EuroQol Valuation Technology
EQ-PVT: EuroQol Portable Valuation Technology
EQ VAS: EuroQol Visual Analogue Scale
HRQoL: Health-related quality of life
QALY: Quality Adjusted Life Year
(c)TTO: (Composite) Time Trade-Off

## Code availability

Code can be shared upon reasonable request to the corresponding author.
